# Squamous cell carcinoma antigen suppresses radiation-induced cell death

**DOI:** 10.1054/bjoc.2000.1683

**Published:** 2001-03

**Authors:** A Murakami, Y Suminami, H Hirakawa, S Nawata, F Numa, H Kato

**Affiliations:** Department of Obstetrics and Gynecology, Yamaguchi University School of Medicine, 1-1-1 Minamikogushi, Ube, 755-8505; Division of Obstetrics and Gynecology, Onoda Municipal Hospital, 1863-1 Higashitakadomari, Onoda, 756-0088, Japan

**Keywords:** SCCA, apoptosis, radiation, caspase 3 activity, caspase 9, p38 MAPK

## Abstract

Previous study has demonstrated that squamous cell carcinoma antigen (SCCA) 1 attenuates apoptosis induced by TNFα, NK cell or anticancer drug. In this study, we have examined the effect of SCCA2, which is highly homologous to SCCA1, but has different target specificity, against radiation-induced apoptosis, together with that of SCCA1. We demonstrated that cell death induced by radiation treatment was remarkably suppressed not only in *SCCA1* cDNA-transfected cells, but also in *SCCA2* cDNA-transfected cells. In these transfectants, caspase 3 activity and the expression of activated caspase 9 after radiation treatment were suppressed. Furthermore, the expression level of phosphorylated p38 mitogen-activated protein kinase (p38 MAPK) was suppressed compared to that of the control cells. The expression level of upstream stimulator of p38 MAPK, phosphorylated MKK3/MKK6, was also suppressed in the radiation-treated cells. Thus, both SCCA1 and SCCA2 may contribute to survival of the squamous cells from radiation-induced apoptosis by regulating p38 MAPK pathway. © 2001 Cancer Research Campaign http://www.bjcancer.com


British Journal of Cancer (2001) 84(6), 851–858
© 2001 Cancer Research Campaign
doi: 10.1054/ bjoc.2000.1683, available online at http://www.idealibrary.com on IDEAL®

### Squamous cell carcinoma antigen suppresses radiation-induced cell death

**A Murakami[1,2], Y Suminami[1], H Hirakawa[1], S Nawata[1], F Numa[1] and H Kato[1]**

[1]Department of Obstetrics and Gynecology, Yamaguchi University School of Medicine, 1-1-1 Minamikogushi, Ube 755-8505; [2]Division of Obstetrics and Gynecology, Onoda Municipal Hospital, 1863-1 Higashitakadomari, Onoda 756-0088, Japan

**Summary** Previous study has demonstrated that squamous cell carcinoma antigen (SCCA) 1 attenuates apoptosis induced by TNFα, NK cell or anticancer drug. In this study, we have examined the effect of SCCA2, which is highly homologous to SCCA1, but has different target specificity, against radiation-induced apoptosis, together with that of SCCA1. We demonstrated that cell death induced by radiation treatment was remarkably suppressed not only in *SCCA1* cDNA-transfected cells, but also in *SCCA2* cDNA-transfected cells. In these transfectants, caspase 3 activity and the expression of activated caspase 9 after radiation treatment were suppressed. Furthermore, the expression level of phosphorylated p38 mitogen-activated protein kinase (p38 MAPK) was suppressed compared to that of the control cells. The expression level of upstream stimulator of p38 MAPK, phosphorylated MKK3/MKK6, was also suppressed in the radiation-treated cells. Thus, both SCCA1 and SCCA2 may contribute to survival of the squamous cells from radiation-induced apoptosis by regulating p38 MAPK pathway. © 2001 Cancer Research Campaign http://www.bjcancer.com

**Keywords**: SCCA; apoptosis; radiation; caspase 3 activity; caspase 9; p38 MAPK

Squamous cell carcinoma antigen (SCCA) was first found in the uterine cervical squamous cell carcinoma (SCC) by Kato and Torigoe (1977). The serum level of SCCA is increased in parallel to the growth of the tumour size or the recurrence of the disease. Therefore, measurement of the serum level of SCCA has been used clinically for the diagnosis and the management of SCC of the uterine cervix as well as other various organs (Kato et al, 1982, 1983; Maruo et al, 1985; Mino et al, 1988; Brioschi et al, 1991).

In a previous report, this tumour marker has been reported to be separated into two fractions (a neutral form and an acidic form) by the column isoelectric focusing (Kato et al, 1984). The netural form was present in both SCC and normal squamous cells with p$I \geq 6.25$, whereas the acidic form, with p$I < 6.25$, was increased mainly in SCC and was released easily into the outside of the cells (Kato et al, 1984). The cDNA of human *SCCA1* gene was first isolated and reported by Suminami et al (1991). Recently, a second *SCCA* gene, *SCCA2*, was identified and was arrayed with *SCCA1* gene on the chromosome 18q21.3 (Kuwano et al, 1995; Schneider et al, 1995). Sequences of *SCCA1* and *SCCA2* cDNAs are highly homologous (95%) and the predicted amino acid sequence analyses indicate that SCCA1 and SCCA2 sequences share 92% identical residues overall with identical secondary structures (Schneider et al, 1995). Two-dimensional analyses of recombinant SCCA1 and SCCA2 indicated that SCCA1 encoded spots with p*I* 6.4, 6.3, 6.0 and 5.9 (two neutral forms and two acidic forms in the previous criteria), whereas SCCA2 encoded spots with p*I* 5.5 and 5.3 (two acidic forms) (Murakami et al, 2000). Quantitative analyses of *SCCA1* and *SCCA2* mRNAs showed that *SCCA2* mRNA expressed in SCC tissues was higher than that in normal tissues, while *SCCA1* mRNA did not show significant differences between them (Murakami et al, 2000).

*Received 4 July 2000*
*Revised 13 December 2000*
*Accepted 15 December 2000*

*Correspondence to:* H Kato; Email: hkato@po.cc.yamaguchi-u.ac.jp

Both SCCA belong to the ovalbumin-serine proteinase inhibitor (ov-serpin) family (Suminami et al, 1991; Remold-O'Donnell, 1993; Schneider et al, 1995). Ov-serpins have unique characteristics which distinguish them from other members of the serpin superfamily. While most serpins are secreted and work extracellulary, ov-serpins are intracellular proteinase inhibitors, which are secreted only occasionally, without a signal sequence, by unknown mechanisms (Belin et al, 1989). Recombinant SCCA1 actually has the inhibitory activities of serine proteinase such as chymotrypsin and cysteine proteinases such as cathepsin L, K, S and papain, while SCCA2 is able to inhibit serine proteinases such as cathepsin G and mast cell chymase in vitro (Nawata et al, 1995, 1997; Sehick et al, 1997, 1998). Therefore, it was speculated that SCCA1 and SCCA2 might have some different biological functions.

Apoptosis, the process of programmed cell death, can be initiated at the cell surface by activation of cell death signals that are transmitted to both the cytoplasm and the nucleus to induce proteolysis and DNA fragmentation (Kerr et al, 1972; Wyllie et al, 1980). Among the apoptotic stimuli, TNF-α, Fas and extracellular stresses such as γ irradiation, UV irradiation, anticancer drugs and osmotic shock activate stress-activated mitogen-activated protein kinase (MAPK) such as p38 and stress-activated protein kinase (SAPK) (Sluss et al, 1994; Xia et al, 1995; Chen et al, 1996a, 1996b, 1999; Brenner et al, 1997).

The p38 MAPK and SAPK are themselves phosphorylated and activated by the upstream kinases. p38 MAPK is activated by phosphorylated MAPK kinase (MKK) 3, MKK4 and MKK6 (Xia et al, 1995; Chen et al, 1999). These upstream kinases are also phosphorylated and activated by further upstream kinases (Hibi et al, 1993; Chen et al, 1999).

Because cancer therapy uses stresses such as radiation therapy and chemotherapy, which induce apoptosis, failure to undergo apoptosis may contribute to the resistance of cancer cells to these therapeutic modalities. For example, many cancer cells express the anti-apoptotic gene *bcl-2*, although increased expression level of Bcl-2 does not always indicate poor prognosis (Furuya et al, 1996; Krajewska et al, 1996). It was reported that intracellular proteinases mediate apoptosis, and on the other hand, proteinase inhibitors are involved in the regulation of apoptosis (Tewari and Dixit, 1995; Tewari et al, 1995a, 1995b; Alnemri et al, 1996). Among the serpin family, Crm A and PAI-2 have been reported to suppress apoptosis in the infection and inflammation processes. Recently we reported that SCCA1 suppressed apoptosis induced by anti-cancer agents TNF-α or natural killer cells in vitro, and *SCCA1* transduced cells grew faster in vivo (Suminami et al, 2000). These data suggest that the existence of SCCA1 makes cancer cells resistant by suppressing apoptosis (Suminami et al, 2000). However, the effect of SCCA2 to apoptosis has been unclear.

In this report, we show that not only SCCA1, but also SCCA2, attenuate radiation-induced apoptosis in vitro. Caspase 3 activity and activation of caspase 9 were suppressed in *SCCA*-transfected cells than those of the control cells. Furthermore, we indicated that SCCA suppresses p38 MAPK pathway in the radiation-induced apoptosis.

### MATERIALS AND METHODS

### Cell lines

A human renal epithelial cell line 293T, which was transformed with adenovirus *E1a* and SV40 large T antigen, was maintained in Eagle's minimal essential medium (EMEM) (Gibco BRL, Gaithersburg) supplemented with 10% fetal calf serum (FCS), 2 mM glutamine, 100 mg $ml^{-1}$ streptomycin, and 100 U $ml^{-1}$ penicillin (Numa et al, 1995). Human uterine cervical cancer cell line SKG IIIa, which expresses SCCA, was kindly provided by Dr S Nozawa (Keio University, Tokyo) (Nozawa et al, 1990). This cell line was grown in Ham's F12 (Dainippon Pharmaceutical, Tokyo) supplemented with 5% FCS, 100 U $ml^{-1}$ penicillin, 100 mg $ml^{-1}$ streptomycin and 2 mM glutamine at 37˚C in a humidified 5% $CO_2$ incubator.

### Construction of the expression vectors

The coding regions of *SCCA1* and *SCCA2* cDNAs were amplified by reverse transcription (RT)-PCR. The first-strand cDNA was generated by incubation of 1 μg of total RNA extracted from SKG IIIa in 20 μl of RT reaction mixture with final concentration of 10 mM Tris-HCl (pH 8.3), 50 mM KCl, 2 mM $MgCl_2$, 1 U $\mu l^{-1}$ RNase inhibitor (Perkin Elmer, Norwalk), 2.5 U $\mu l^{-1}$ MuLV reverse transcriptase (Perkin Elmer), 1 mM dNTPs and 2.5 μM of Oligo d $(T)_{16}$ (Perkin Elmer). The incubation was performed at 42˚C for 15 min followed by heat-inactivation of the enzyme at 99˚C for 5 min.

Following RT reaction, PCR was performed in 50 μl of mixture with final concentration of 10 mM Tris-HCl (pH 8.3), 50 mM KCl, 5 mM $MgCl_2$, 0.2 mM dNTPs, 0.025 U $\mu l^{-1}$ of Ampli Taq DNA polymerase (Perkin Elmer) and the following primers: Sense primer, 5′-TCACCATGAATTCACTCAG-3′; antisense primer, 5′-TCTATGGGGATGAGAATCT-3′. The reaction was performed at 94˚C for 2 min followed by 30 cycles of 94˚C for 1 min, 58˚C for 30 s and 72˚C for 2 min, and final extension at 72˚C for 10 min. The RT-PCR products were ligated with pCR II vector (Invitrogen, Carlsbad) and the nucleotide sequences of the ligated products (pCR II-SCCA1 and pCR II-SCCA2) were confirmed by the dideoxy nucleotide chain termination method using automated DNA sequencing apparatus of LI-COR 4000LS (LI-COR Inc, Lincoln).

The *Xho*I fragments of pCR II-SCCA1 and pCR II-SCCA2 containing full length of coding region of *SCCA1* and *SCCA2* cDNAs were ligated with *Xho*I site of the eukaryote expression vector pCEP4 (Invitrogen) and the direction was confirmed. These constructs (pCEP4-SCCA1 and pCEP4-SCCA2) and the control construct without *SCCA* cDNA were used for the transfection.

### Gene transfection

Cultured 293T cells were plated at density of approximately $3 \times 10^5$ cells per 35 mm dish (Becton Dickinson, Foster City). On the day of transfection, the cultured cells were washed with Opti-MEM medium (Gibco BRL) and transfected with 2.5 μg of pCEP4-SCCA1, pCEP4-SCCA2, or the vector alone by the poly-amine transfection method (TransIT-LT1, Pan Vera, Madison), according to the manufacturer's instructions. Cells were cultured in EMEM containing 10% FCS and 200 μg $ml^{-1}$ hygromycin B (Sigma, St. Louis) for 6 weeks of incubation for selecting the independent clones (SCCA1: 293T-SCCA1-1 and 293T-SCCA1-2; SCCA2: 293T-SCCA2-1 and 293T-SCCA2-2).

### Expression of the SCCA cDNA in the transfected cells

The expression of SCCA1 and SCCA2 in parent cells and hygromycin-resistant cells were confirmed by semi-quantitative RT-PCR. It was performed with RNA-PCR core kit (Perkin Elmer) according to the manufacture's protocol with amplifying primer pair for *SCCA1* and *SCCA2* cDNA (sense primer, 5′-GTTGGATCCAACAAGCTCTTCGGAGA-3′; antisense primer, 5′-CCGTCGACTCTACGGGGATGAGAATCT-3′) or L-19 amplifying primer pair (sense primer, 5′-CTGAAGGTCAAAGG-GAATGTG-3′; antisense primer, 5′-GGACAGAGTCTTGATG ATCTC-3′). RT reaction was performed with 1 μg of total RNA and RT condition was described above. PCR was done with 28 cycles (confirmed in the preliminary experiments to be in the exponential phase) of 94˚C, 58˚C, and 72˚C each for 1 min, by adding 0.1 μl of [α-$^{32}$P] dCTP. Radioactivity of the PCR bands were counted with Bioimaging analyser BAS 2000 (Fuji Film, Tokyo) and the ratio of the counts obtained with the SCCA1 or SCCA2, and L19 was calculated.

The transfected cells were resuspended in ice-cold PBS, sonicated mildly, and next centrifuged at 15 000 rpm for 10 min at 4˚C. The expression level of SCCA protein in cell lysate of the transfected cells was confirmed by IMx system (DaiNabot, Tokyo) (Takeshima et al, 1990).

### Cell growth assay

The transfected cells were seeded on a 96-well plate ($2 \times 10^3$ cells $well^{-1}$). After 1, 3 and 5 days, 150 μl of 3-[4,5-dimethylthiazol-2-yl]-2, 5-diphenyltetrazolium bromide (MTT) (0.5 mg $ml^{-1}$ in culture medium) was added per well, and the incubation was continued for a further 4 h. The medium was then aspirated, and the cells were dissolved in 150 μl of dimethyl sulfoxide (DMSO) and the absorbance at 540 nm was measured with a microplate reader (Toso, Tokuyama).

 © 2001 Cancer Research Campaign

### Radiation treatment

The transfected cells were seeded on 96-well plate ($5 \times 10^3$ cells $well^{-1}$). After 24 h of incubation, these cells were exposed to the radiation at 0, 5 or 20 Gy. Incubation was continued for another 36 h, and cells were collected and used for the detection of mRNAs and proteins. Viability of cells was determined at 12, 24 and 36 h of incubation by MTT assay as described above.

### Expression levels of bax and bcl-2 mRNAs

Total cellular RNAs were extracted using RNeasy kit (Quiagen, Hilden) according to the manufacturer's protocol. Semi-quantitative RT-PCR was performed using RNA-PCR core kit (Perkin Elmer) following the manufacturer's protocol with the *bcl-2* or *bax* primer pairs: *bcl-2*, 5′-GACTTCGCCGAGATGTCCAG-3′ (sense primer) and 5′-TCACTTGTGGCTCAGATAGG-3′ (antisense primer); *bax*, 5′-GGTTTCATCCAGGATCGAGACGG-3′ (sense primer) and 5′-ACAAAGATGGTCACGGTCTGCC-3′ (antisense primer).

RT reaction was performed with 1.0 μg of total RNA and RT condition was described above. PCR was done with 28 cycles (confirmed in the preliminary experiments to be in the exponential phase) of 94˚C for 1 min, 60˚C for 1 min and 72˚C for 1.5 min for amplifying *bcl-2* mRNA, and 94˚C for 1 min, 50˚C for 1 min and 72˚C for 2 min for amplifying *bax* mRNA, by adding 0.1 μl of [α-$^{32}$P] dCTP. Radioactivity of each PCR band was counted with Bioimaging analyser BAS 2000 and the ratio of the obtained counts with the *bcl-2* or *bax*, and *L19* were calculated.

### Detection of caspase 2, 8 and 9 expression and caspase 3 activity

Caspase 2, caspase 8, and caspase 9 were detected by SDS-polyacrylamide gel electrophoresis (PAGE) and the immunochemical technique. Fifty-five μg of total protein in each lane was electrophoresed on 12.5% polyacrylamide gel. After SDS-PAGE was completed, proteins were transferred to the polyvinylidene difluoride (PVDF) membrane (ATTO, Tokyo) with semi-dry type blotting. The transferred membranes were stained by immunochemical technique which consisted of the following procedure: after blocking the membrane with blocking solution (5% skimmed milk with 0.1% Tween 20 dissolved in Tris buffered saline, pH 7.5), the blotted membranes were incubated with polyclonal antibody against caspase 2 [caspase-2 (C-20)-G], caspase 8 [Mch 5 p20 (T-16)] (SantaCruz Biotechnology, Delaware), or caspase 9 (New England Biolabs, Beverly), which were diluted in blocking solution (1:2000). Then these membranes were incubated with peroxidase conjugated rabbit anti-goat IgG antibody (caspase 2 and caspase 8) or swine anti-rabbit IgG antibody (caspase 9) diluted in the blocking solution. Finally, ECL-Western blotting detection system (Amersham, Aylesburg) was applied according to the manufacture's protocol and then the membranes were exposed to the hyperfilm-ECL (Amersham).

Caspase 3 activity was measured by using ApoAlert CPP32 protease assay kit (Clontech, Palo Alto) according to the manufacture's protocol. The treated cells were harvested, counted, lysed with the cell lysis buffer and centrifuged. After incubation with the substrate for 1 h at 37˚C, the caspase 3 activity was obtained by measuring $OD_{400}$.

### Expression levels of p38 MAPK and SAPK/JNK

Approximately $1 \times 10^6$ of the irradiated cells were collected in one tube and Laemmli's loading buffer was added to the collected cells and the cells were mildly sonicated. After sonication, products were boiled for 5 min and were centrifuged at 15 000 rpm for 15 min at 4˚C. The treated samples of the same volume were electrophoresed on 12.5% polyacrylamide gel. After SDS-PAGE was completed, proteins were transferred to the PVDF membrane and blocking procedures were performed as described above.

The membranes were treated as follows: after blocking the membrane in the blocking solution, immunoblotting was done to detect p38 MAPK, phosphorylated p38 MAPK, phosphorylated MKK3/MKK6 and phosphorylated SAPK/JNK using p38 MAPK, phospho-p38 MAPK (Thr180/Tyr182), phospho-MKK3/MKK6 (Ser189/207) and phospho-SAPK/JNK (Thr183/Tyr185) antibodies, respectively (New England Biolabs). Then the membranes were incubated with peroxidase conjugated swine anti-rabbit IgG antibody (p38 MAPK, phospho-p38 MAPK and phospho-MKK3/MKK6) or rabbit anti-mouse IgG antibody (phospho-SAPK/JNK), and developed as described above.

### Statistical analysis

Statistical analysis was done by Duncan's new multiple range test. A probability value of $< 0.05$ was considered to be significant.

### RESULTS

### Establishment of the expressing clones of SCCA1 and SCCA2

To analyse the functions of the SCCA in tumour cells, we established the stable clones expressing SCCA. A human kidney cell line 293T, which did not express SCCA, was transfected with eukaryotic expression vector containing *SCCA1*, *SCCA2* cDNA or vector alone as a control cell. To examine the expression levels of the SCCA in these transfected cells and the parent cell line, semi-quantitative RT-PCR of *SCCA* mRNA was performed. The cells transfected with *SCCA* cDNA expressed remarkably higher levels of *SCCA* mRNA, while the control and the parent cells did not express *SCCA* mRNA (Figure 1).

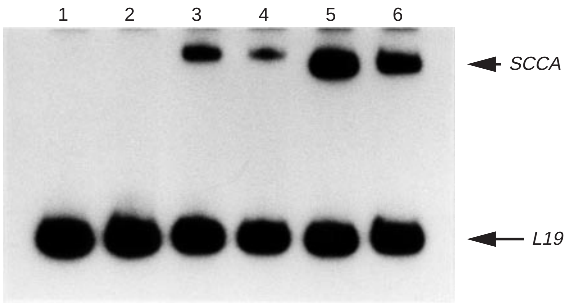

**Figure 1** Expression of SCCA1 and SCCA2 in 293T cells transfected with *SCCA1*, *SCCA2* cDNA or vector alone. Expression levels of *SCCA1* and SCCA2 mRNAs were determined by semi-quantitative RT-PCR. The upper arrowhead indicates *SCCA1* and *SCCA2*. The lower arrowhead indicates *L19* as an internal control. Lane 1 = 293T; Lane 2 = 293T-pCEP4; Lane 3 = 293T-SCCA1-1; Lane 4 = 293T-SCCA1-2; Lane 5 = 293T-SCCA2-1; Lane 6 = 293T-SCCA2-2

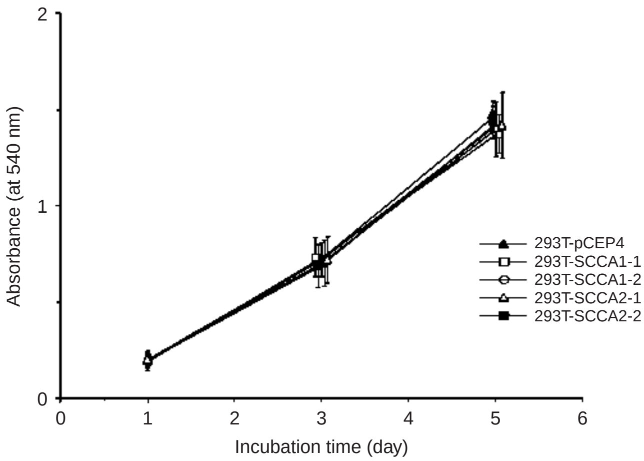

**Figure 2** Cell growth rate in 293T cells transfected with *SCCA1*, *SCCA2* cDNA or vector alone. There were no significant differences in the cell growth rate among every clone. These data were obtained in six independent experiments and indicated as mean ± SEM

### Effect of SCCA1 and SCCA2 on the viability after irradiation

The growth rates of these transfected cells were evaluated with MTT assay. However, there were no differences in the rate of cell growth by the transfection of SCCA (Figure 2). We then investigated the role of SCCA in the apoptosis induced by radiation. The viable cells were measured by MTT assay until 36 h after exposure to different doses of radiation. There were significant differences in the viability between *SCCA* cDNA transfected clones and the control cells irradiated with 5 Gy (Figure 3A) and 20 Gy (Figure 3B and Figure 4).

### Detection of caspase 2, 8 and 9 expression and caspase 3 activity

We evaluated the expression levels of *bcl-2* and *bax* mRNAs in the *SCCA* cDNA-transfected cells, the control cells, and the parent cells. Each cell expressed *bcl-2* and *bax* mRNAs and there were no significant differences in the basal expression levels among these cells. Furthermore, these expression levels were not altered by the expression of SCCA1 and SCCA2 even after irradiation with 5 Gy or 20 Gy, as measured by semi-quantitative RT-PCR (data not shown).

We then examined the expression levels of caspase 2, 8, and 9 with Western blotting and caspase 3 activity before and after radiation treatment. As shown in Figure 5A, there were no significant differences in the expression level of caspase 8 and caspase 2 between the control cells and the cells transfected with *SCCA* cDNAs. On the other hand, increase of the activated form of caspase 9 after radiation treatment was suppressed in the *SCCA*-transfected cells when compared to the control cells. Caspase 3 activity was also suppressed in the cells transfected with *SCCA1* or *SCCA2* cDNA (293T-SCCA1-1 or 293T-SCCA2-1) than that of the control cells (293T-pCEP4) (Figure 5B). There were no significant differences in the expression levels of caspase 2, 8, 9 and caspase 3 activity between the control cells and SCCA transfectants 12 and 24 h after irradiation treatment (data not shown).

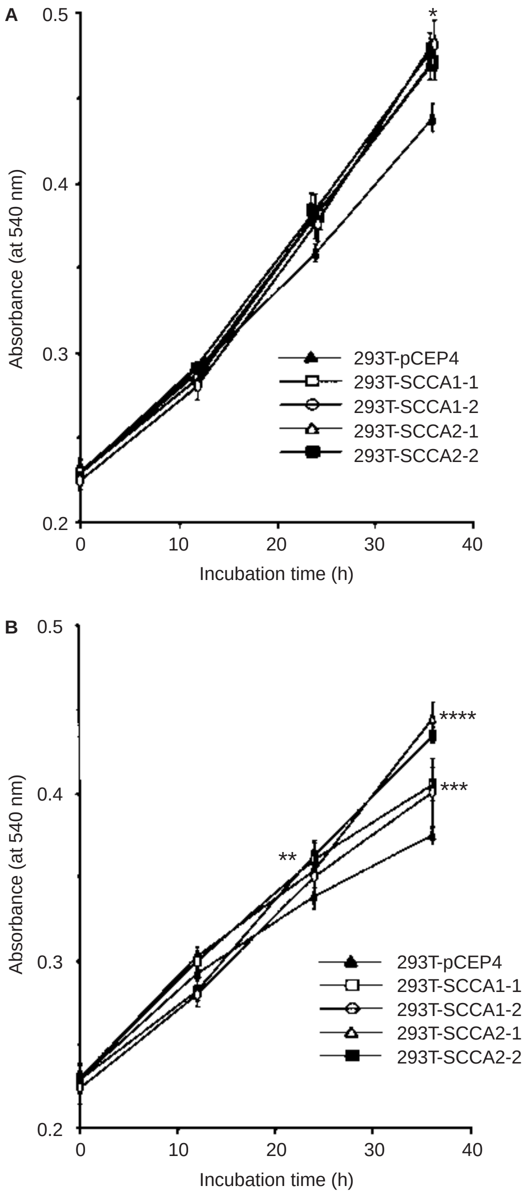

**Figure 3** Cell viability of *SCCA* cDNA transfectants after radiation. Cells were cultured after radiation treatment with 5 Gy (**A**) or 20 Gy (**B**) for indicated periods of time as described in 'Materials and methods', and the viable cells were determined by MTT assay. These data were obtained in six independent experiments and indicates as means ± SEM (**P* < 0.05 for 293T-pCEP4 vs other clones; ***P* < 0.05 for 293T-pCEP4 vs *SCCA2* cDNA transfectants; ****P* < 0.05 for 293T-pCEP4 vs SCCA1 cDNA transfectants; *****P* < 0.01 for 293T-pCEP4 vs *SCCA2* cDNA transfectants)

### Expression levels of p38 MAPK and SAPK/JNK

The apoptosis induced by stress like UV or irradiation has been reported to induce phosphorylation of MAPK. We examined the expression levels of p38 MAPK and SAPK/JNK by Western blotting. As shown in Figure 6, there were no significant differences in the expression levels of total p38 MAPK among these cells. However, expression level of phosphorylated p38 MAPK of *SCCA* cDNA-transfected cells (293T-SCCA1-1 and 293T-SCCA2-1) were significantly less than that of the control cells before radiation treatment. When these cells were treated with radiation 36 h after treatment, *SCCA1* cDNA-transfected cells (293T-SCCA1-1) showed a slight increase of phosphorylated p38

 *© 2001 Cancer Research Campaign*

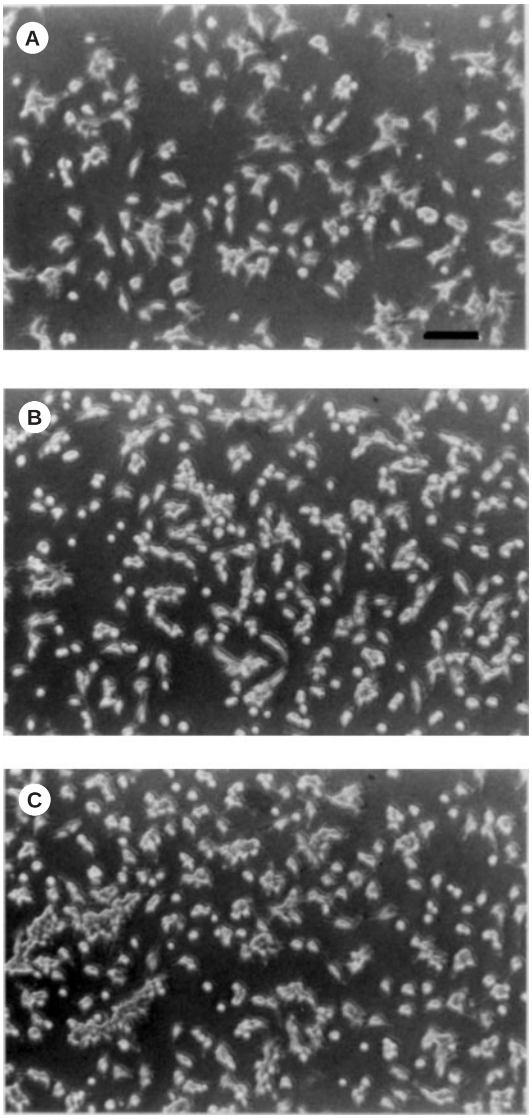

**Figure 4** The viable cells with radiation treatment after 36 h in the control cells and the *SCCA* cDNA transfectants. The cells were treated with 20 Gy of radiation and observed using phase contrast microscopy. (**A**) 293T-pCEP4, (**B**) 293T-SCCA1-1, (**C**) 293T-SCCA2-1. Scale bar = 100 µm

MAPK, although it was still less than that of the control cells (293T-pCEP4). In the case of *SCCA2* cDNA-transfected cells (293T-SCCA2-1), the cells were resistant to the increase of phosphorylated p38 MAPK. The activator of p38 MAPK (phosphorylated MKK3/MKK6) was present before the radiation treatment in each cell. However, the expression level was decreased 36 h after radiation treatment in *SCCA*-transfected cells, although it was increased in the control cells. We could not find the differences of the expression levels of phosphorylated p38 MAPK and MKK3/MKK6 between the control cells and the *SCCA* transfectants at 12 and 24 h (data not shown). On the other hand, there were no significant differences in the expression levels of SAPK/JNK and phosphorylated SAPK/JNK among the control cells and *SCCA* transfectants with radiation treatment (data not shown).

### DISCUSSION

SCCA (SCCA1 and SCCA2) belongs to the ov-serpin family. Some of the ov-serpin family has been reported as inhibiting apoptosis. For example, PAI-2 can inhibit the apoptosis induced by TNF-α or Fas (Enari et al, 1995; Tewari and Dixit, 1995) and PI-9 can inhibit granzyme B-induced apoptosis (Bird et al, 1998). Furthermore, CrmA is able to inhibit apoptosis by suppressing caspase 3 (Wagenknecht et al, 1998). Recently we reported that SCCA1 significantly suppressed the apoptosis induced by anti-cancer agent, TNF-α, (or activated natural killer (NK) cells (Suminami et al, 2000).

It has not been known how SCCA2 acts inside and/or outside of the cell, although it has high homology with SCCA1. In a previous report, it was suggested that SCCA2 may regulate inflammation and tissue degradation by cathepsin G and mast cell chymase within the epithelia of the skin because of the inhibition of these proteinases in vitro (Schick et al, 1997). We demonstrated that when SCCA was genetically engineered to express in 293T cells, which did not express SCCA in the cell, apoptosis induced by radiation treatment was significantly suppressed. This data demonstrated that expression of SCCA2 in tumour cells, as well as SCCA1, might contribute to the defence system of tumour cells, protecting them from apoptotic cell death induced by radiation.

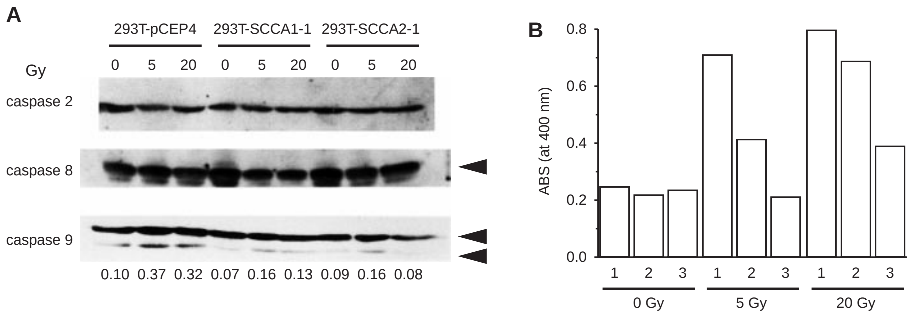

**Figure 5** Expression of caspases in the irradiated cells after 36 h. (**A**) Expression of caspase 2, 8 and 9 in the control cells and *SCCA* cDNA transfectants with or without radiation treatment. In the middle panel, arrowhead indicates activated caspase 8. Upper and lower arrowheads indicate procaspase 9 and activated caspase 9, respectively. Activation of caspase 9 was evaluated as a ratio of activated form and procaspase which was obtained by densitometric analysis and indicated below each lane. (**B**) Caspase 3 activity in the control cells and *SCCA* cDNA transfectants with or without radiation treatment. Lane 1 = 293T-pCEP4; Lane 2 = 293T-SCCA1-1; Lane 3 = 293T-SCCA2-1. These results are representative of three independent experiments

© 2001 Cancer Research Campaign

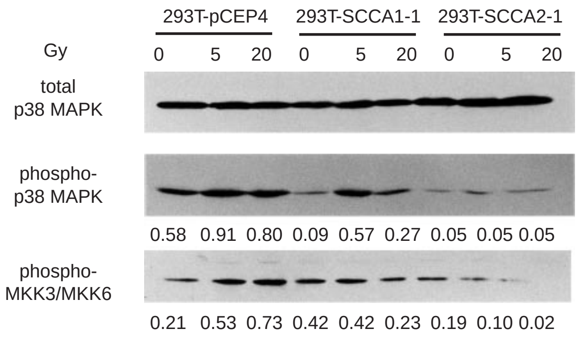

**Figure 6** Expression of p38 MAPK and MKK3/MKK6 in the control cells and the *SCCA* cDNA transfectants with or without radiation treatment after 36 h. Expression levels of kinases of p38 MAPK signals were determined by Western blot analyses. Activation of p38 MAPK is indicated as a ratio of densitometric intensity of phosphorylated form and total p38 MAPK. The ratio of phosphorylated MKK3/MKK6 and total p38MAPK is also indicated. These results are representative of three independent experiments

Recently, a number of physiologic inhibitors of apoptosis have been identified, including the Bcl-2 family members, inhibitor of apoptosis protein family and the serpin family. Bcl-2 is one of the most familiar inhibitors of apoptosis that is induced by various stimuli. It prevents apoptosis; on the other hand Bax opposes the function of Bcl-2 and hence promotes apoptosis. Bcl-2 binds with Bax, prevent its homodimerzation and thus whether or not apoptosis occurs. It was reported that radiation treatment has been shown to induce increases in *bax* mRNA in several cells (Zhan et al, 1994). Miyashita et al (1994) demonstrated that the expression level of Bcl-2 was down-regulated by p53. To know the mechanism of inhibition of radiation-induced apoptosis by SCCA, we first analysed the expression levels of *bax* and *bcl-2* mRNAs and p53 protein in *SCCA1* or *SCCA2* cDNA-transfected cells. However, they were not affected by the expression of SCCA. Furthermore, there were no significant differences in the expression levels of *bax* and *bcl-2* mRNAs after radiation treatment between the *SCCA1* or *SCCA2* cDNA-transfected cells and the control cells. Therefore, in this study Bcl-2 and Bax did not affect the regulation of cell death by SCCA.

As some caspases sequentially process and activate others, a model has been proposed in which some caspases (such as caspase 2, 8 or 9) act as initiator or signalling proteinases, while caspase 3 acts as an effector of apoptosis (Cohen, 1997). Previous studies have suggested that caspase 9 is the most upstream member of the apoptotic proteinases (Li et al, 1997). Caspase 3 is the most essential proteinase for the nuclear changes associated with apoptosis, including chromatin condensation, and caspase 9 was the most important participant for caspase 3 activation (Li et al, 1997). These findings point to the existence of an apoptotic pathway dependent on both caspase 9 and caspase 3. However, recent studies suggested that comparison of the requirement for caspase 9 and caspase 3 in different apoptotic settings indicates the existence of at least four different apoptotic pathways in mammalian cells (Hakem et al, 1998). Our study suggested that, in 293T cells, radiation preferentially triggers the activation of an apoptotic pathway involving caspase 9 and caspase 3 activity and both SCCAs act in this apoptotic cascade.

The MAPK family plays an important role in cell growth, differentiation, transformation and apoptosis (Cobb and Goldsmith, 1995; Karin, 1995; Xia et al, 1995; Dickens et al, 1997; Hunter, 1997). Recently several studies revealed that SAPK/JNK activation and/or p38 MAPK activation were involved in apoptosis induced by different stimuli, such as radiation, UV and osmotic shock-induced apoptosis (Sluss et al, 1994; Xia et al, 1995; Chen et al, 1996a, 1996b, 1999; Brenner et al, 1997). Consistently, blockade of JNK activation promotes cell survival (Park et al, 1996; Dickens et al, 1997). Although some reports suggest that SAPK/JNK and/or the p38 MAPK pathways function primarily to promote apoptotic cell death, different reports showing controversial results are observed in some systems (Natoli et al, 1995; Lenczowski et al, 1997; Nishina et al, 1997; Yang et al, 1997).

Some reports propose that the duration and magnitude of p38 MAPK activation are major determinants of the cells fate (Guo et al, 1998; Takekawa and Sato, 1998). Thus, the biochemical regulation and cellular role of MAPK family in apoptosis cascades are still to be clarified. In the present study, we examined whether radiation stress induced phosphorylated SAPK/JNK and phosphorylated p38 MAPK, which were activated forms. In this study, p38 MAPK was less phosphorylated in the *SCCA* cDNA-transfected cells, not only after radiation treatment but also before treatment. Furthermore, phosphorylated MKK3/MKK6, which is the active form of MAPK kinase and works just upstream of p38 MAPK, was suppressed after radiation in the *SCCA* cDNA-transfected cells, while that in the control cells was increased after radiation. Therefore, in this system, p38 MAPK is proapoptotic, and SCCA1 and SCCA2 attenuate apoptosis by suppressing p38 MAPK signalling at the step of phosphorylation of p38 MAPK and/or MKK3/MKK6, although it is still unclear whether it affects the phosphorylation directly or indirectly.

In this study, radiation-mediated apoptosis induces activation of caspase 9, caspase 3 activity, and phosphorylation of MKK3/MKK6 and p38 MAPK. The relationship between the signal stream of caspases and the pathway of p38 MAPK has been controversial. Now, there are three evidences in this relationship: 1. the activation of caspases was required for the activation of p38 MAPK (Natoli et al, 1997); 2. p38 MAPK stimulated the activity of caspases (Nemoto et al, 1998; Zhang et al, 2000); 3. the early stage of JNK and p38 MAPK activation was a caspase-independent pathway, but the late phase activation of them was a caspase-independent pathway (Roulston et al, 1998). As we have not analysed the effects of the inhibitors of p38 MAPK or caspases, we can't decide which one locates upstream in our system. However, we speculate that p38 MAPK pathway locates upstream of caspases, and SCCA affects the p38 MAPK pathway first, inhibiting caspase 9 as a secondary effect, because phosphorylated p38 MAPK was suppressed in both SCCA transfectants without radiation treatment.

Recently it has been reported that serine proteinase inhibitor, N-tosyl-phenylalanine chloromethyl ketone (TPCK), inhibits apoptosis induced by Taxol, through the inhibition of the phosphorylation of c-Raf-1 and Bcl-2 (Huang et al, 1999). However, to our best knowledge, this is the first report that serpin inhibits the phosphorylation of MAPK signalling pathway. We speculate that SCCA1 and SCCA2 indirectly regulate the phosphorylation by inhibiting different factors which are involved in the p38 MAPK pathway because SCCA1 and SCCA2 have different P1-P1′ amino acids which decide the target proteinases of serpin. The genes of *SCCA1* and *SCCA2* are tandemly located on chromosome 18q21.3 and they are thought to be made as a result of gene duplication. Therefore, although their target molecules may be different, it is reasonable that they have similar functions.

 *© 2001 Cancer Research Campaign*

We previously reported that the expression level of *SCCA2* mRNA in cancer tissues was higher than that of normal tissues (Murakami et al, 2000). However, it has been unclear why and for what role SCCA2 increases in the SCC tissues. Although we have already analysed the promoter region of the *SCCA2* gene (Sakaguchi et al, 1999), more detailed analysis would explain what molecule stimulates the expression of SCCA2 in the case of malignancy. Recently it has been reported that cathepsin G released from neutrophil stimulates granzyme B of NK cells (Yamazaki and Aoki, 1998; Bird, 1999). Our preliminary data indicate that secreted SCCA1 inhibits chemoinvasion of NK cells. As SCCA2 inhibits the cathepsin G in vitro, not only intracellular SCCA2 but also secreted SCCA2 may augment the inhibitory function of SCCA1 to apoptosis and be necessary for malignant tumours.

In conclusion, our results suggested that both SCCAs inhibited the apoptotic pathways induced by radiation. Overexpression of SCCA in tumour cells appears to be in part responsible for the protection of the cells from apoptosis in vitro and may be profitable for the cancer cells to protect them from the therapeutic modalities.

### ACKNOWLEDGEMENTS

We thank Dr Shiro Nozawa for providing SKG IIIa cells. This study was supported in part by a Grant-in-Aid for Scientific Research on Priority Areas (A) from the Ministry of Education, Science, Sports and Culture, and by a Grant-in-Aid for the Second Term Comprehensive 10-year Strategy for Cancer Control from the Ministry of Health and Welfare, Japan.

### REFERENCES

Alnemri ES, Livingston DJ, Nicholson DW, Selveson G, Thornberry NA, Wong WW and Yuan J (1996) Human ICE/CED-3 protease nomenclature [letter]. *Cell* **87**: 171

Belin D, Wohlwend A, Schleuning WD, Kruithof EK and Vassalli JD (1989) Facultative polypeptide translocation allows a single mRNA to encode the secreted and cytosolic forms of plasminogen activators inhibitor 2. *EMBO J* **8**: 3287–3294

Bird PI (1999) Regulation of pro-apoptotic leucocyte granule serine proteinases by intracellular serpins. *Immunol Cell Biol* **77**: 47–57

Bird CH, Sutton VR, Sun J, Hirst CE, Novak A, Kumar S, Trapani JA and Bird PI (1998) Selective regulation of apoptosis: the cytotoxic lymphocyte serpin proteinase inhibitor 9 protects against granzyme B-mediated apoptosis without perturbing the Fas cell death pathway. *Mol Cell Biol* **18**: 6387–6398

Brenner B, Koppenhoefer U, Weinstock C, Linderkamp O, Lang F and Gulbins E (1997) Fas- or ceramide-induced apoptosis is mediated by a Rac1-regulated activation of Jun N-terminal kinase/p38 kinases and GADD153. *J Biol Chem* **272**: 22173–22181

Brioschi PA, Bischof P, Delafosse C and Krauer F (1991) Squamous-cell carcinoma antigen (SCC-A) values related to clinical outcome of pre-invasive and invasive cervical carcinoma. *Int J Cancer* **47**: 376–379

Chen YR, Meyer CF and Tan TH (1996a) Persistent activation of c-Jun N-terminal kinase 1 (JNK1) in γ radiation-induced apoptosis. *J Biol Chem* **271**: 631–634

Chen YR, Wang X, Templeton D, Davis RJ and Tan TH (1996b) The role of c-Jun N-terminal kinase (JNK) in apoptosis induced by ultraviolet C and γ radiation. *J Biol Chem* **271**: 31929–31936

Chen Z, Seimiya H, Naito M, Mashima T, Kizaki A, Dan S, Imaizumi M, Ichijo H, Miyazono K and Tsuruo T (1999) ASK mediates apoptotic cell death induced by genotoxic stress. *Oncogene* **18**: 173–180

Cobb MH and Goldsmith EJ (1995) How MAP kinase are regulated. *J Biol Chem* **270**: 14843–14846

Cohen GM (1997) Caspases: the executioners of apoptosis. *Biochem J* **326**: 1–16

Dickens M, Rogers JS, Cavanagh J, Raitano A, Xia Z, Halpern JR, Greenberg ME, Sawyers CL and Davis RJ (1997) A cytoplasmic inhibitor of the JNK signal transduction pathway. *Science* **277**: 693–696

Enari M, Hug H and Nagata S (1995) Involvement of an ICE-like protease in Fas-mediated apoptosis. *Nature* **375**: 78–81

Furuya Y, Krajewski S, Epstein JI and Issacs JT (1996) Expression of bcl-2 and the progression of human and rodent prostatic cancers. *Clin Cancer Res* **2**: 389–398

Guo YL, Baysal K, Kang B, Yang LJ and Williamson JR (1998) Correlation between sustained c-Jun N-terminal protein kinase activation and apoptosis induced by tumour necrosis factor-α in rat mesangial cells. *J Biol Chem* **273**: 4027–4034

Hakem R, Hakem A, Duncan GS, Henderson JT, Woo M, Soengas MS, Elia A, Pompa JL, Kagi J, Khoo W, Potter J, Yoshida R, Kaufman SA, Lowe SW, Penninger JM and Mak TW (1998) Differential requirement for caspase 9 in apoptotic pathways in vivo. *Cell* **94**: 339–352

Hibi M, Lin A, Smeal T, Minden A and Karin M (1993) Identification of an oncoprotein- and UV-responsive protein kinase that binds and potentiates the c-Jun activation domain. *Genes Dev* **7**: 2135–2148

Huang Y, Sheikh MS, Fornace AJ Jr and Holbrook NJ (1999) Serine protease inhibitor TPCK prevents Taxol-induced cell death and blocks c-Raf-1 and Bcl-2 phosphorylation in human breast carcinoma cells. *Oncogene* **18**: 3431–3439

Hunter T (1997) Oncoprotein networks. *Cell* **88**: 333–346

Kato H and Torigoe T (1977) Radioimmunoassay for tumour antigen of human cervical squamous cell carcinoma. *Cancer* **40**: 1621–1628

Kato H, Morioka H, Tsutsui H, Aramaki S and Torigoe T (1982) Value of tumour-antigen (TA-4) of squamous cell carcinoma in predicting the extent of cervical cancer. *Cancer* **50**: 1294–1296

Kato H, Tamai K, Morioka H, Nagai M, Nagaya T and Torigoe T (1983) Prognostic significance of the tumour antigen TA-4 in squamous cell carcinoma of the uterine cervix. *Am J Obstet Gynecol* **145**: 350–354

Kato H, Nagaya T and Torigoe T (1984) Heterogeneity of a tumour antigen TA-4 of squamous cell carcinoma in regulation to its appearance in circulation. *Gann* **75**: 433–435

Karin M (1995) The regulation of AP-1 activity by mitogen-activated protein kinases. *J Biol Chem* **270**: 16483–16486

Kerr JFR, Wyllie AH and Currie AR (1972) Apoptosis: basic biological phenomenon with wide-ranging implications in tissue kinetics. *Br J Cancer* **26**: 239–257

Krajewska M, Krajewski S, Epstein JI, Shabaik A, Sauvageot J, Song K, Kitada S and Reed JC (1996) Immunohistochemical analysis of bcl-2, bax, bcl-X, and mcl-1 expression in prostate cancers. *Am J Pathol* **148**: 1567–1576

Kuwano A, Kondo I, Kishi F, Suminami Y and Kato H (1995) Assignment of the squamous cell carcinoma antigen locus (SCC) to 18q21 by in situ hybridization. *Genomics* **30**: 626

Lenczowski JM, Dominguez L, Eder AM, King LB, Zacharchuk CM and Ashwell JD (1997) Lack of a role for Jun kinase and AP-1 in Fas-induced apoptosis. *Mol Cell Biol* **17**: 170–181

Li P, Nijhawan D, Budihardjo I, Srinivasula SM, Ahmad M, Alnemri ES and Wang X (1997) Cytochrome c and dATP-dependent formation of Apaf-1/Caspase-9 complex initiates an apoptotic protease cascade. *Cell* **91**: 479–489

Maruo T, Shibata K, Kimura A, Hoshina A and Mochizuki M (1985) Tumour-associated antigen, TA-4, in the monitoring of the effects of therapy for squamous cell carcinoma of the uterine cervix. *Cancer* **59**: 302–308

Mino N, Iio A and Hamamoto K (1988) Availability of tumour-antigen 4 as a marker of squamous cell carcinoma of the lung and other organs. *Cancer* **62**: 730–734

Miyashita T, Krajewski S and Krajewska M (1994) Tumour suppressor p53 is a regulator of *bcl-2* and *bax* gene expression *in vitro* and *in vivo*. *Oncogene* **9**: 1799–1805

Murakami A, Suminami Y, Sakaguchi Y, Nawata S, Numa F, Kishi F and Kato H (2000) Specific detection and quantitation of *SCC antigen 1* and *SCC antigen 2* mRNAs by fluorescence-based asymmetric semi-nested reverse transcription PCR. *Tumour Biol* **21**: 224–234

Nawata S, Tsunaga N, Numa F, Tanaka T, Nakamura K and Kato H (1995) Serine protease inhibitor activity of recombinant squamous cell carcinoma antigen towards chymotrypsin, as demonstrated by sodium dodecyl sulfate-polyacrylamide gel electrophoresis. *Electrophoresis* **16**: 1027–1030

Nawata S, Nakamura K, Tanaka T, Numa F, Suminami Y, Tsunaga N, Kakegawa H, Katsunuma N and Kato H (1997) Electrophoretic analysis of the ‘cross-class’ interaction between novel inhibitory serpin, squamous cell carcinoma antigen-1 and cystein proteinases. *Electrophoresis* **18**: 784–789

Natoli G, Costanzo A, Ianni A, Templeton DJ, Woodgett JR, Balsano C and Levrero M (1995) Activation of SAPK/JNK by TNF receptor 1 through a noncytotoxic TRAF2-dependent pathway. *Science* **275**: 200–203

Nemoto S, Xiang J, Huang S and Lin A (1998) Induction of apoptosis by SB 202190 through inhibition of p38β mitogen-activated protein kinase. *J Biol Chem* **273**: 16415–16420

Nishina H, Fischer KD, Radvanyi L, Shahinian A, Hakem R, Rubie EA, Bernstein A, Mark TW, Woodgett JR and Penninger JM (1997) Stress-signalling kinase Sek1 protects thymocytes from apoptosis mediated by CD95 and CD3. *Nature* **385**: 350–353

Nozawa S, Kojima M, Tukazaki K, Sakayori M, Iizuka R and Kagiyama N (1990) *In vitro* and *in vivo* induction of squamous cell carcinoma antigen (SCC) in a uterine cervical cancer cell line (SKG IIIa) with peplomycin and sodium butyrate. *Asia-Oceania J Obstet Gynecol* **16**: 153–160

Numa F, Hirabayashi K, Tsunaga N, Kato H, O'Rourke K, Shao H, Stechmann-Lebakken C, Varani J, Rapraeger A and Dixit VM (1995) Elevated levels of Syndecan-1 expression confer potent serum-dependent growth in human 293T cells. *Cancer Res* **15**: 4676–4680

Park DS, Stefanis L, Yan CYI, Farinelli SE and Greene LA (1996) Ordering the cell death pathway. *J Biol Chem* **271**: 21898–21905

Remold-O'Donnell E (1993) The ovalbumin family of serpin proteins. *FEBS Lett* **315**: 105–108

Roulston A, Reinhard C, Amiri P and Williams LT (1998) Early activation of c-Jun N-terminal kinase and p38 kinase regulate cell survival in response to tumour necrosis factor α. *J Biol Chem* **273**: 10232–10239

Sakaguchi Y, Kishi F, Murakami A, Suminami Y and Kato H (1999) Structural analysis of human SCC antigen 2 promoter. *Biochim Biophys Acta* **1444**: 111–116

Schick C, Kamachi Y, Bartuski AJ, Çataltepe S, Schechter NM, Pemberton PA and Silverman GA (1997) Squamous cell carcinoma antigen 2 is a novel serpin that inhibits the chymotrypsin-like proteinases cathepsin G and mast cell chymase. *J Biol Chem* **17**: 1849–1855

Schick C, Pemberton PA, Shi GP, Kamachi Y, Çataltepe S, Bartuski AJ, Cornstein ER, Brömme D, Chapman HA and Silverman GA (1998) Cross-class inhibition of the cysteine proteinases cathepsin K, L, S by the serpin squamous cell carcinoma antigen 1: A kinetic analysis. *Biochemistry* **37**: 5258–5266

Schneider SS, Schick C, Fish KE, Miller E, Pena JC, Treter SD, Hui SM and Silverman GA (1995) A serine proteinase inhibitor locus at 18q21.3 contains a tandem duplication of the human squamous cell carcinoma antigen gene. *Proc Natl Acad Sci USA* **92**: 3147–3151

Sluss HK, Barrett T, Derijard B and Davis RJ (1994) Signal transduction by tumour necrosis factor mediated by JNK protein kinases. *Mol Cell Biol* **14**: 8376–8384

Suminami Y, Kishi F, Sekiguchi K and Kato H (1991) Squamous cell carcinoma antigen is a new member of the serine protease inhibitors. *Biochem Biophys Res Commun* **181**: 51–58

Suminami Y, Nagashima S, Vujanovic NL, Hirabayashi K, Kato H and Whiteside TL (2000) Inhibition of apoptosis in human tumour cells by the tumour-associated serpin, SCC antigen-1. *Br J Cancer* **82**: 981–989

Takekawa M and Saito H (1998) A family of stress-inducible GADD45-like proteins mediate activation of the stress-responsive MTK1/MEKK4 MAPKKK. *Cell* **95**: 521–530

Takeshima N, Nakamura K, Takeda O, Morioka H, Tamura H, Takasugi N and Kato H (1990) Individualization of the cutoff value for squamous-cell carcinoma antigen using sensitive enzyme immunoassay. *Tumour Biol* **11**: 167–172

Tewari M and Dixit VM (1995) Fas- and tumour necrosis factor-induced apoptosis is inhibited by the poxvirus crmA gene product. *J Biol Chem* **270**: 3255–3260

Tewari M, Quan LT, O'Rourke K, Desnoyers S, Zeng Z, Beidler DR, Poirier GG, Salveson GS and Dixit VM (1995a) Yama/CPP 32 beta, a mammalian homolog of CED-3, is a CrmA-inhibitable protease that cleaves the death substrate poly (ADP-ribose) polymerase. *Cell* **81**: 801–809

Tewari M, Telford WG, Miller RA and Dixit VM (1995b) CrmA, a poxvirus-encoded serpin, inhibits cytotoxic T-lymphocyte-mediated apoptosis. *J Biol Chem* **270**: 22705–22708

Wagenknecht B, Schulz JB, Gulbins E and Weller M (1998) Crm-A, bcl-2 and NDGA inhibit CD95-L-induced apoptosis of malignant glioma cells at the level of caspase 8 processing. *Cell Death Diff* **5**: 894–900

Wyllie AH, Kerr JFR and Currie AR (1980) Cell death: the significance of apoptosis. *Int Rev Cytol* **68**: 251–306

Xia Z, Dickens M, Raingeaud J, Davis RJ and Greenberg ME (1995) Opposing effects of ERK and JNK-p38 MAP kinases on apoptosis. *Science* **270**: 1326–1331

Yamazaki T, Aoki Y (1998) Cathepsin G enhances human natural killer cytotoxicity. *Immunology* **93**: 115–121

Yang X and Khosravi-Far R, Chang HY and Baltimore D (1997) Daxx, a novel Fas-binding protein that activates JNK and apoptosis. *Cell* **89**: 1067–1076

Zhan Q, Fan S, Bae I, Guillof C, Liebermann DA, O'Connor PM and Fornance Jr AJ (1994) Induction of bax by genotoxic stress in human cells correlates with normal p53 status and apoptosis. *Oncogene* **9**: 3743–3751

Zhang J, Gao J.-X, Salojin K, Shao Q, Grattan M, Meagher C, Laird DW and Delovitch TL (2000) Regulation of Fas ligand expression during activation-induced cell death in T cells by p38 mitogen-activated protein kinase and c-Jun NH2-terminal. *J Exp Med* **191**: 1017–1029

 © 2001 Cancer Research Campaign
